# Portal vein thrombosis in a patient on semaglutide

**DOI:** 10.5339/qmj.2025.57

**Published:** 2025-06-11

**Authors:** Mohammed F. Farooqi, Maria Khan, Muhammad Arshad, Adnan Agha

**Affiliations:** 1 Department of Internal Medicine, Tawam Hospital, Al-Ain, United Arab Emirates; 2 Department of Internal Medicine, College of Medicine and Health Sciences, United Arab Emirates University, Al-Ain, United Arab Emirates; 3 Department of Diabetes & Endocrinology, Tawam Hospital, Al-Ain, United Arab Emirates *Email: adnanagha@uaeu.ac.ae

**Keywords:** Obesity, semaglutide, thrombophilia, type 2 diabetes mellitus, weight loss, venous thromboembolism

## Abstract

**Background:** Obesity and type 2 diabetes mellitus (T2DM) are both modern-day pandemics, significantly impacting worldwide healthcare. The glucagon-like peptide-1 receptor agonist (GLP1-RA) semaglutide is a novel treatment for both T2DM and obesity; however, it can be associated with an increased risk of venous thromboembolism (VTE).

**Case presentation:** This case report describes a 59-year-old woman with T2DM who received semaglutide for the management of glycemic levels and also experienced the additional advantage of weight reduction. Within 6 months of initiating GLP1-RA, the patient presented with lower back pain associated with nausea and poor oral intake. She had no known risk factors for VTE or thrombophilia or any history of significant illness in her family. Her physical examination revealed no significant findings; only mild leukocytosis and neutrophilia were detected. She underwent an abdominal computed tomography scan, which revealed intrahepatic portal vein thrombosis without evidence of liver cirrhosis or abdominal malignancy. Her symptoms improved with oral anticoagulation (rivaroxaban). The result of the thrombophilia examination was negative for inherited or acquired thrombophilia, with the exception of mutation of Janus kinase 2, which may increase the risk of thrombosis.

**Conclusions:** The use of GLP1-RA is increasing due to the growing desire for weight loss medications; therefore, it is pertinent for physicians to have a better understanding of the possible risks for thrombosis before initiating GLP1-RA treatment.

## INTRODUCTION

Obesity is defined as the excessive accumulation of adipose tissue, either centrally or in the subcutaneous areas. Obesity is classified using the body mass index (BMI), which is calculated as the weight divided by the height squared, as follows: normal, 18.5–24.9 kg/m^2^; overweight, 25–29.9 kg/m^2^; and obese, ≥30 kg/m^2^.^1^ The World Health Organization declared obesity a healthcare crisis in 2017, with over two billion adults being overweight, and 30% of them are obese.^2^ Glucagon-like peptide-1 (GLP-1) is a hormone secreted by L cells in the ileum upon meal stimulation. GLP-1 induces insulin secretion in relation to a meal with a low risk of hypoglycemia.^3^ Long-acting GLP1 receptor agonists (GLP1-RAs) are potent glucose-lowering agents and are effective at reducing weight.^3^ The GLP-RA semaglutide is 94% similar in structure to GLP-1, with three substitutions to prolong its half-life. Semaglutide is administered as a weekly subcutaneous injection in patients with type 2 diabetes mellitus (T2DM) and/or obesity.^4^ Patients treated with semaglutide have achieved glycemic control and weight loss in recent clinical trials, with 5%–10% weight loss achieved in most patients.^5^ In the present case, a woman with T2DM and obesity was treated with semaglutide and developed portal vein thrombosis (PVT).

## CASE PRESENTATION

A 59-year-old woman with a history of T2DM presented to the emergency department with dull lower back pain that started after excessive household cleaning for 3 days. Other than this pain, the patient complained of nausea and poor oral intake. The pain was not relieved by paracetamol. The patient denied any vomiting, diarrhea, changes in bowel habits, or urinary symptoms. She had previously had three healthy children with no history of venous thromboembolism (VTE), abortions, or thrombophilia. The patient had no significant family history other than parents with DM. The patient was not treated with hormone replacement therapy and never smoked or drank alcohol. Her medications included injectable semaglutide 1 mg once weekly (Thursdays), which she had started 4 months ago for diabetes control and had lost 5 kg of weight while taking this treatment.

Physical examination revealed the following: afebrile; heart rate, 80 beats/minute; blood pressure, 146/80 mmHg; oxygen saturation, 98% on room air; and respiratory rate, 17/min. She weighed 75 kg with a BMI of 31.5 kg/m^2^. Her physical examination revealed no significant abnormalities, with no costal angle tenderness, calf tenderness, or abdominal findings. However, the patient continued to complain of lower back pain, occasional moderate right quadrant pain, and nonresolving nausea. Her laboratory investigations only revealed mild leukocytosis and neutrophilia with normal liver and renal function tests (Table 1).

**Figure 1:**
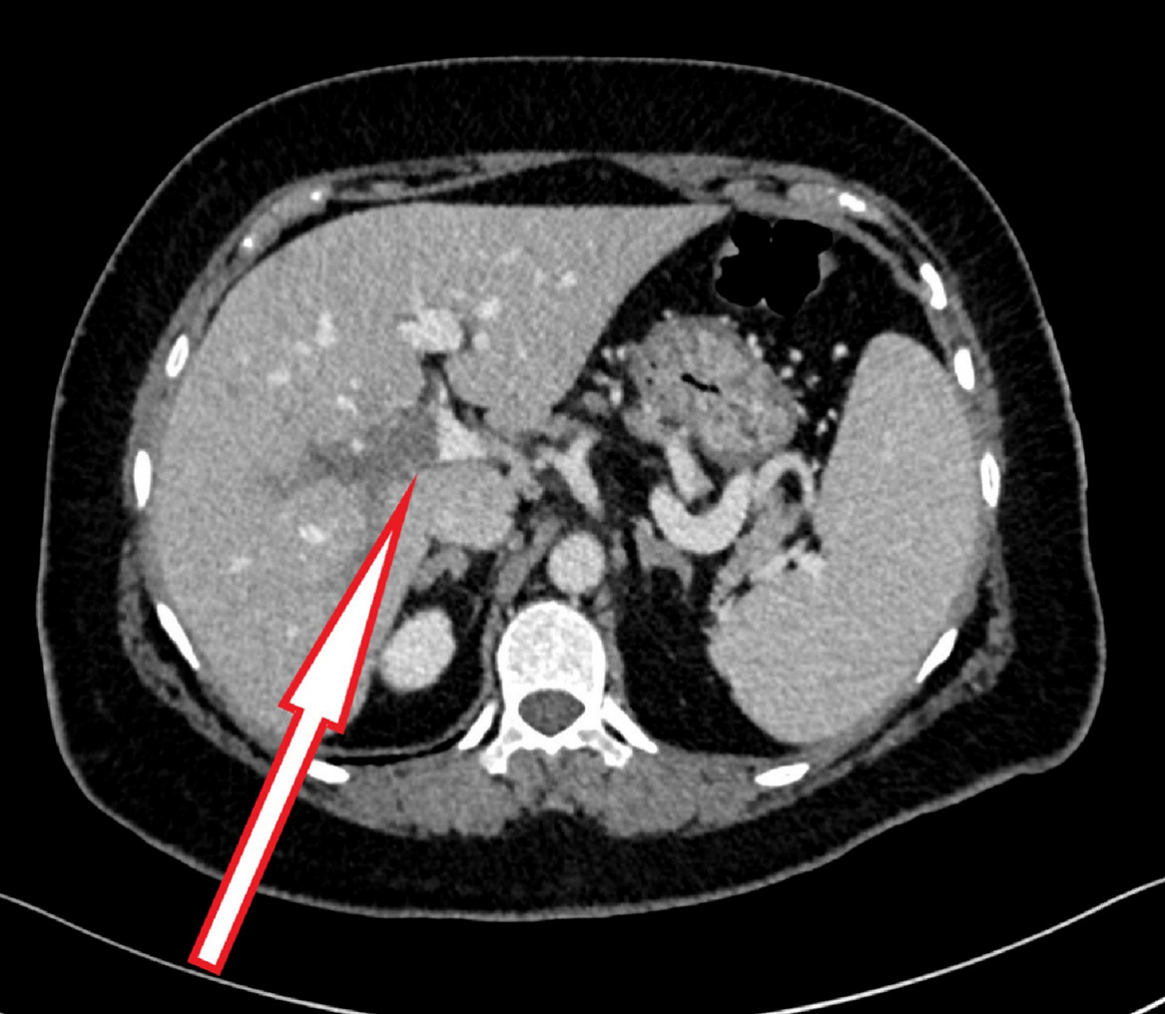
Abdominal CT scan showing intrahepatic portal vein thrombosis (arrow).

**Figure 2:**
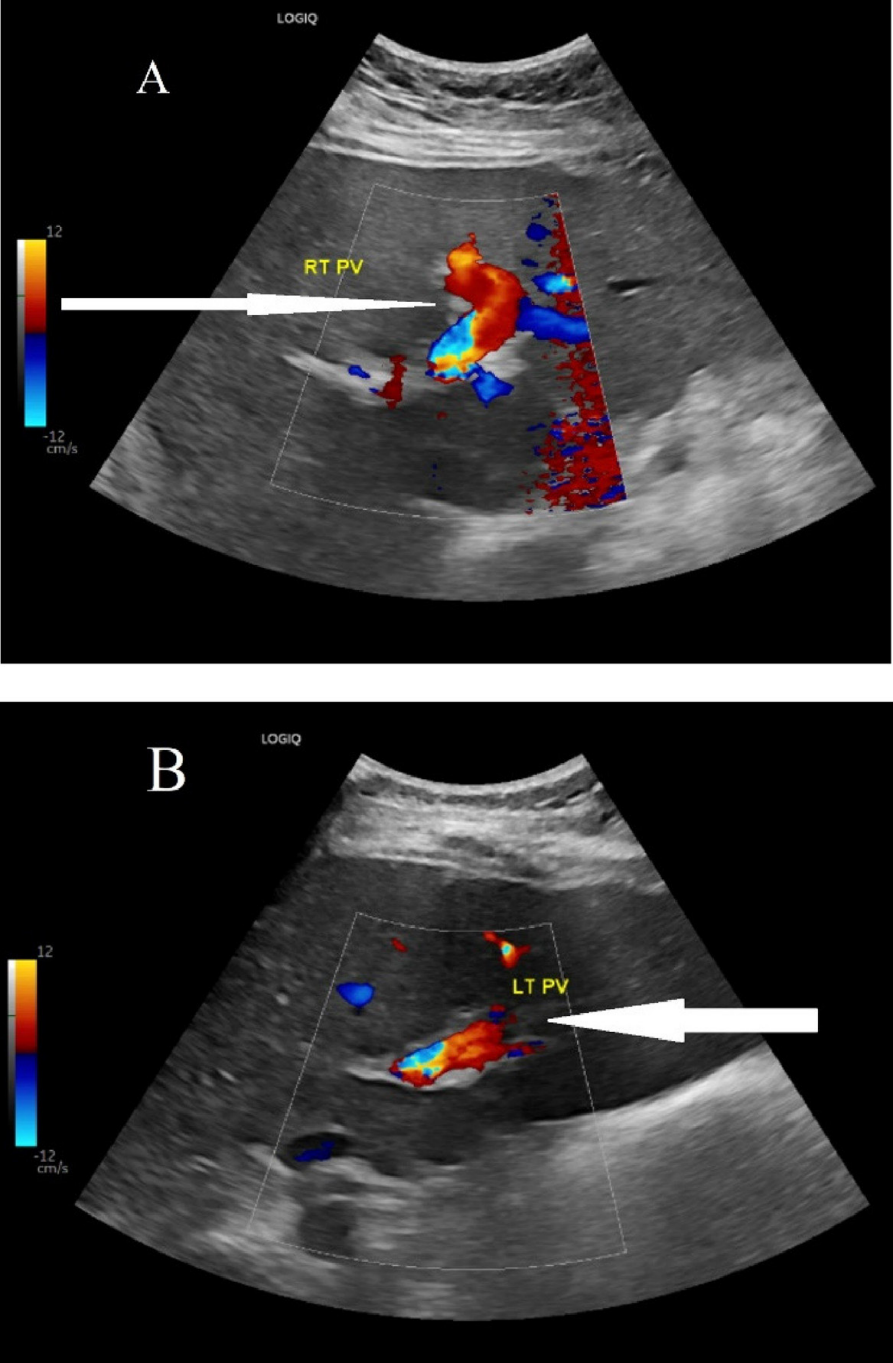
Abdominal ultrasound with liver doppler of portal veins revealing good blood flow seen in (A) right portal vein indicated by long white arrow and good blood flow seen in (B) left portal vein indicated by short white arrow.

A contrast-enhanced computed tomography (CT) scan was performed to identify the source of the pain and rule out any obstructive gastrointestinal cause. The CT scan revealed evidence of intrahepatic PVT involving the right branch and part of the left branch, but there was no evidence of collection or a mass (Figure 1). An upper esophagogastroduodenoscopy revealed mild gastritis. The patient was treated with pantoprazole for gastritis for 2 weeks, and oral anticoagulation (rivaroxaban) therapy was initiated. The patient exhibited clinical improvement within 2 weeks with increased appetite and reduced back pain and was discharged with a hematology follow-up to investigate the cause of thrombophilia. The hematology findings did not reveal any specific causes of inherited or acquired thrombophilia, although the patient had a Janus kinase 2 (JAK2) mutation, which may increase the risk of thrombosis (Table 2). An abdominal ultrasound at the 3-month follow-up showed recanalization of the thrombosed portal vein (Figure 2). The patient received anticoagulation treatment for 6 months with complete resolution of symptoms.

## DISCUSSION

PVT is caused by thrombosis of the main portal vein or one of its branches, with or without extension into the mesenteric or splenic vein. PVT is categorized based on etiology as malignant or benign. Benign PVT usually develops in patients with portal hypertension secondary to liver cirrhosis and rarely occurs in patients with healthy livers.^6^ Benign PVT is rare, with an annual incidence of approximately four cases per 100,000 in the normal population.^7^ However, the incidence of benign PVT increases in patients with liver cirrhosis to up to 11%.^8^ Benign PVT can be acute or chronic, based on the presence of collateral circulation and the thrombus characteristics. Chronic PVT does not require treatment with anticoagulants.^9^ Up to 85% of patients with healthy liver status and benign PVT have an acquired or inherited prothrombotic condition.^10,11^ A recent meta-analysis in Asian individuals with benign PVT and healthy livers demonstrated that thrombophilia was caused by protein C deficiency (10.7%), JAK2 mutations (8.5%), and antiphospholipid antibodies in 7.5%.^12^ The JAK-2 V617F-mutation, a transversion mutation at nucleotide 1849 of JAK2, results in a valine-to-phenylalanine substitution at codon 617 and is associated with an increased risk of venous thrombosis via endothelial over-expression of the von Willebrand factor and p-selectin. This mutation occurs in up to one-third of patients with benign PVT and healthy livers.^13,14^ A proportion of patients with JAK2 mutations develop myeloproliferative neoplasms (MPN), characterized by the proliferation of stem cells such as mature granulocytes (primary myelofibrosis), red blood cells (polycythemia vera), and/or platelets (essential thrombocythemia).^15^

Our patient had a JAK-2 mutation with no evidence of MPN. This mutation may have contributed to the development of PVT. Obesity is a common risk factor for VTE,^16^ and although our patient had this risk factor, no evidence suggested that this condition contributed to the PVT. A recent meta-analysis also identified the use of GLP1-RA as a risk factor for VTE. Patients treated with semaglutide have a 266% (relative risk of 3.66) increased risk of developing VTE, leading to concerns about its suitability for use in individuals already at risk of VTE.^17^ Recent literature has also reported superior mesenteric thrombosis in a patient taking dulaglutide,^18^ another weekly GLP1-RA, but to the best of the authors’ knowledge, there are no reports of PVT associated with GLP1-RA. The recent advancements and increased availability of long-acting GLP1-RA resulted in their widespread use, causing even more concerns about adverse events. In our patient, semaglutide initiation coincided with the development of PVT. Although the co-occurrence of PVT and the initiation of semaglutide could be incidental, the patient had a JAK-2 mutation without any overt MPN. A literature search revealed a similar case report; a 57-year-old male patient with T2DM taking the potent long-acting GLP1-RA, dulaglutide, developed mesenteric vein thrombosis. This case highlights a rare but significant adverse event associated with GLP1-RA and the need to carefully observe patients for unusual complications when initiating such therapy.

## CONCLUSION

Our case report describes a healthy middle-aged woman with no previous risk factors for thrombophilia who developed PVT within 6 months of initiating GLP1-RA for weight loss and T2DM management. A JAK2 mutation without any evidence of MPN was identified in the patient. Based on this case report, healthcare professionals should be aware of the risks of VTE and PVT. An unusual clinical presentation of PVT may be associated with GLP1-RA. Thus, an appropriate risk assessment should be performed before initiating such treatment. It is important to remain vigilant in identifying any signs and symptoms of VTE and/or PVT in these patients and to educate them accordingly. This case report also reiterates the importance of ensuring that appropriate hematology input is sought early and that thrombophilia screening, including testing for JAK2 mutation, is performed whenever a patient presents with benign PVT and a healthy liver. This case report is the first in the region to document an incidence of benign PVT in a patient with a JAK2 mutation and treated with semaglutide.

## Compliance with ethical standards

None.

## Conflicts of interest

All authors declare no conflicts of interest.

## Human rights statement

Informed consent was obtained from the patient, and ethical approval was obtained from the Human Research Ethics Committee (MF2058-2024-1067).

## Figures and Tables

**Table 1.** Laboratory investigations on admission.

Laboratory test	Value	Normal range
Sodium	141	136–146 mmol/L
Potassium	3.6	3.6–5.1 mmol/L
Chloride	104	98–107 mmol/L
Bicarbonate	21	22–29 mmol/L
Creatinine	60	45–84 µmol/L
Urea	3.4	2.67–8.07 mmol/L
Albumin	36	>21 g/L
Calcium	2.33	2.23–2.58 mmol/L
Amylase	105	28–100 units/mL
Lipase	45	13–60 IU/L
Aspartate aminotransferase	24	<32 IU/L
Alanine aminotransferase	32	<33 IU/L
Hemoglobin	143	117–155 g/L
Mean corpuscular volume	81.7	81–100 fL
C-reactive protein	37.9	<5 mg/L

**Table 2.** Laboratory investigations for thrombophilia.

Thrombophilia investigations	Results	Normal range
PT	12.1 seconds	9.5–12.5 seconds
INR	1.13	0.87–1.15
APTT	27.5 seconds	22.2–34.2 seconds
Fibrinogen	4.41 g/L	1.5–3.87 g/L
Factor V	71%	70%–120%
Antithrombin III	101%	80%–120%
Protein C act	104%	70%–130%
Protein S free	76%	50%–134%
D-dimer	1.64 g/L	0.129–0.523 mg/L
Cardiolipin IgG	<2.6 CU	<20 CU
Cardiolipin IgM	5.4 CU	<20 CU
B2 glycoprotein IgG	<6.4 CU	<20 CU
B2 glycoprotein IgM	<1.1 CU	<20 CU
MDx factor V Leiden	Not detected	
MDx factor II (prothrombin)	Not detected	
MDx MTHFR	Heterozygous	
MDx JAK2 (V617F) mutation PCR	Detected	
Homocysteine total	9 µmol/L	12–15 µmol/L
Flow cytometry report	Negative for PNH	

PT: prothrombin time, APTT: activated partial thromboplastin clotting time, INR: international normalized ratio, B2: beta-2, Ig: Immunoglobulin, JAK: Janus kinase, PCR: polymerase chain reaction, MTHFR: methylenetetrahydrofolate, PNH: paroxysmal nocturnal hemoglobinuria.
